# Activation of CTU2 expression by LXR promotes the development of hepatocellular carcinoma

**DOI:** 10.1007/s10565-024-09862-9

**Published:** 2024-04-17

**Authors:** Chao Xue, Zhuo Wei, Ye Zhang, Ying Liu, Shuang Zhang, Qi Li, Ke Feng, Xiaoxiao Yang, Guangqing Liu, Yuanli Chen, Xiaoju Li, Zhi Yao, Jihong Han, Yajun Duan

**Affiliations:** 1 College of Life Sciences, Key Laboratory of Medicinal Chemical Biology, Key Laboratory of Bioactive Materials of Ministry of Education, Nankai University, Tianjin, China; 2 Tianjin Institute of Obstetrics and Gynecology, Tianjin Key Laboratory of Human Development and Reproductive Regulation, Tianjin Central Hospital of Obstetrics and Gynecology, Tianjin, China; 3 Guizhou Medical University, Guiyang, China; 4 Key Laboratory of Metabolism and Regulation for Major Diseases of Anhui Higher Education Institutes, Anhui Provincial International Science and Technology Cooperation Base for Major Metabolic Diseases and Nutritional Interventions, College of Food and Biological Engineering, Hefei University of Technology, Hefei, China; 5 Key Laboratory of Immune Microenvironment and Disease of the Ministry of Education, Department of Immunology, School of Basic Medical Sciences, Tianjin Medical University, Tianjin, China; 6 Department of Cardiology, The First Affiliated Hospital of USTC, Division of Life Sciences and Medicine, University of Science and Technology of China, Hefei, China

**Keywords:** HCC, CTU2, LXR, Lipogenesis, Cell proliferation

## Abstract

Cytosolic thiouridylase 2 (CTU2) is an enzyme modifying transfer RNAs post-transcriptionally, which has been implicated in breast cancer and melanoma development. And we found CTU2 participated in hepatocellular carcinoma (HCC) progression here. HepG2 cells as well as xenograft nude mice model were employed to investigate the role of CTU2 in HCC development in vitro and in vivo respectively. Further, we defined CTU2 as a Liver X receptor (LXR) targeted gene, with a typical LXR element in the CTU2 promoter. CTU2 expression was activated by LXR agonist and depressed by LXR knockout. Interestingly, we also found CTU2 took part in lipogenesis by directly enhancing the synthesis of lipogenic proteins, which provided a novel mechanism for LXR regulating lipid synthesis. Meanwhile, lipogenesis was active during cell proliferation, particularly in tumor cells. Reduction of CTU2 expression was related to reduced tumor burden and synergized anti-tumor effect of LXR ligands by inducing tumor cell apoptosis and inhibiting cell proliferation. Taken together, our study identified CTU2 as an LXR target gene. Inhibition of CTU2 expression could enhance the anti-tumor effect of LXR ligand in HCC, identifying CTU2 as a promising target for HCC treatment and providing a novel strategy for the application of LXR agonists in anti-tumor effect.

**Graphical Abstract:**

1.) CTU2 enhances proliferation of hepatoma carcinoma cells.

2.) CTU2 is the target gene of LXR, and LXR can transcriptionally activate CTU2 expression.

3.) CTU2 can promote protein synthesis of lipogenic genes.

4.) Inhibiting CTU2 expression can synergistically enhance the inhibitory effects of LXR ligands on HCC growth.

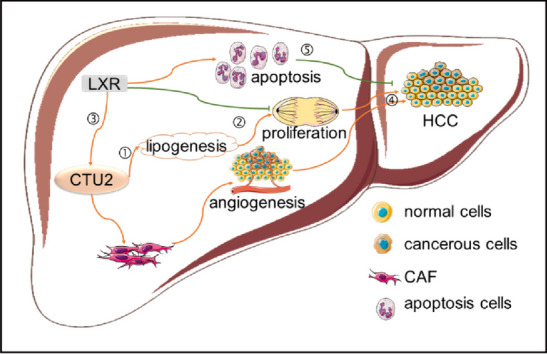

**Supplementary Information:**

The online version contains supplementary material available at 10.1007/s10565-024-09862-9.

## Introduction

HCC is considered one of the most common tumors and a leading cause of cancer-related mortality worldwide (Miller et al. 2022). There are many risk factors for the development of HCC, such as HBV and HCV infections, alcohol abuse, oxidative stress, hyperlipidemia, diabetes, and obesity (Athavale et al. 2018; Chouhan et al. 2016; Chouhan et al. 2020; Toh et al. 2023). The most effective treatment of HCC is physical excision of the pathological tissue and alternative treatment is not valid to improve the patient’s prognosis once the tumor is unresectable (Bruix et al. 2004). Therefore, exploring the mechanisms regulating HCC progression is of great importance due to its fatality rate and poor diagnosis.

Liver X receptors (LXRs) are ligand-activated transcription regulators, including two isoforms, LXRα (Nr1H3) and LXRβ (Nr1H2). Oxysterols, such as 25-hydroxycholesterol (25HC), function as endogenous LXR ligands (Bovenga et al. 2015). LXR activity is modulated by synthetic ligands, such as T0901317 and GW3965 (Buñay et al. 2021). LXRs have been implicated in a variety of malignancies, and their ligands have been shown to induce pyroptosis and inhibit inflammatory responses and proliferation in tumor cells (Derangere et al. 2014; Lin and Gustafsson 2015). However, LXR regulates de novo lipogenesis by transcriptional activation of its target genes such as the transcriptional regulatory element sterol-regulatory element binding protein 1 (SREBP1) and lipid synthase fatty acid synthase (FASN), stearoyl-CoA desaturase 1 (SCD1), and acetyl-CoA carboxylase 1 (ACC1) (Currie et al. 2013). De novo lipogenesis is active during embryonic development and prominent in the lactating breast and the cycling endometrium of adults but is low in most other normal human tissues (Kuhajda 2000). Aberrant lipogenesis is involved in diabetes, obesity, metabolic syndrome as well as cancer (Menendez and Lupu 2007). Enhanced lipogenesis is important for cancer cells because it provides lipids for membrane building blocks, post-translational modification of proteins, and energy storage (Menendez and Lupu 2007). At the molecular level, increased mRNA levels of FASN, ACC1, and SCD1 have been determined in human HCC (Yahagi et al. 2005). SREBP1 overexpression is also associated with HCC development while inversely correlates with HCC patients’ prognosis (Li et al. 2017). In addition, suppression of FASN is found to be detrimental for HCC growth (Gao et al. 2006). However, whether LXR-enhanced lipid synthesis affects its antitumor activity is unknown.

The gene encoding cytosolic thiouridylase 2 (CTU2) is located on Chromosome 16. CTU2 is important in the post-transcriptional modification of transfer RNAs (tRNAs). CTU2 binds with the conserved CTU1 protein, and the complex takes part in the 2-thiolation of cytosolic tRNAs. It is proposed that thiolation of the wobble base and methoxycarbonylmethyl (mcm5S2U) can regulate base pairing with purines, thereby improving codon reading accuracy (Numata et al. 2006). tRNA thiolation takes part in the maintenance of genome integrity, growth under nutritionally challenging environments (Laxman et al. 2013), and root development (Philipp et al. 2014). In addition, CTU2 is elevated in breast tumors and promotes metastasis through supporting specific translation of oncogenic factor LEF1 in an internal ribosome entry site (IRES) dependent manner (Delaunay et al. 2016). Besides, one study reveals that CTU2-linked tRNA modification promotes melanoma growth by regulating HIF1α codon-dependent translation (Rapino et al. 2018). However, if CTU2 is involved in HCC remains unclear. Herein, we identified CTU2 as an LXR target and determined the role of CTU2 in HCC development.

## Methods

### Reagents

Sigma-Aldrich's MTT assay kit was used. Matrigel basement membrane matrix was purchased from Corning (Bedford, MA, USA). Dual-Luciferase Reporter Assay kit was obtained from Promega (Madison, WI, USA). T0901317 (Cayman Chemical, Michigan, USA) dissolved in DMSO was used in vitro, and equal volume of DMSO as control. 25HC (Sigma-Aldrich, USA) was dissolved in ethanol and ethanol as control. Lipofectamine® 2000 was purchased from Invitrogen (Carlsbad, CA, USA). Triglyceride content detection kit and Nile red staining kit was purchased from Solarbio (Beijing, China). Antibody information was listed in Supplementary Table S3.

### Cell lines and plasmids

HepG2 cells were obtained and cultured as described (Wang et al. 2020). To generate cDNA for human CTU2, the lentiviral vector (pLV)-CTU2 expression vector (pLV-CTU2) was constructed by primers listed in Supplementary Table S1. shRNA against CTU2 (shCTU2) and SREBP1 (shSREBP1) were subcloned into pLKO.1-TCR, respectively, and shRNA oligo sequences were listed in Supplementary Table S1. The lentiviral particles were prepared and used to infect target cells to obtain stably overexpressing or knockdown cells. LXRα and LXRβ cDNAs were subcloned into pEGFP-C2 vector as described (Chen et al. 2012). The LXRα or LXRβ deficient HepG2 cells were obtained as previously described with the guide oligo sequences listed in Supplementary Table S1 (Liu et al. 2018).

### Xenograft tumor model

Cells were collected and mixed with isopyknic matrigel basement membrane matrix. About 1 × 10^6^ cells were injected subcutaneously (s.c.) into the flank side of nude mice (6-week-old, male, BALB/c Foxn1^nu^/ Nju, GemPharmatech, Jiangsu, China). When tumors were visible, mice were given either regular chow or chow with T0901317 [5 mg/day/kg bodyweight (mpk)] for 2 weeks based on the previous study (Wang et al. 2016). Tumor volumes were routinely assessed at the indicated time points. Mice were sacrificed, followed by collecting liver and tumor samples. Livers were photographed and a piece of liver was used for triglyceride (TG) analysis. Tumors were weighed, photographed, and used for other assays.

### Dual-luciferase report assay

pGL-CTU2 vector was constructed according to primers listed in Table S1, and CTU2mut in Fig. 2o was constructed as described (Ma et al. 2015). Using Lipofectamine® 2000 regent, pGL-CTU2 promoters and *Renilla* DNA were transfected into HepG2 cells. After 24-h transfection and indicated treatment, a dual luciferase report assay was performed as described (Liu et al. 2018). Each sample's promoter activity was defined as the ratio of promoters’ luciferase activity to *Renilla*, and then it was normalized to each control group, which was defined as 1.

### cDNA synthesis and qRT-PCR

Following the extraction of RNA from cells, cDNA was synthesized (Ma et al. 2015). qRT-PCR was performed and the sequence of primers was listed in Supplementary Table S2. Target gene mRNA levels were normalized by GAPDH mRNA.

### Western blot, immunofluorescent staining, and immunohistochemistry staining

Cell proteins were isolated as described following the indicated treatment (Chen et al. 2015). Protein expression of GAPDH, CTU2, pre-SREBP1, n-SREBP1, ACC1, and FASN was detected by Western blot.

The 5-μm paraffin or frozen sections were prepared and processed with immunohistochemistry or immunofluorescent staining of CTU2, Ki67, VEGFA, αSMA, IFNγ, FASN, Nile red, or TUNEL staining (Yang et al. 2019).

### Oil red O staining

Cells were cultured on coverslips in a 12-well plate (~ 5*10^5^/well), followed by fixed with paraformaldehyde. Cells were stained with Oil Red O solution (0.3% Oil Red O in 60% isopropanol) for 60 min and then washed twice with water. After soaking in water for five minutes, cells were stained with hematoxylin solution for two minutes.

### MTT assay

HepG2 cells (~ 5*10^4^/well) were plated in a 96-well plate. After cultured for 24 h, each well was added with 20 μL MTT solution (1 mg/mL in serum-free medium). After 4 h incubation, MTT was aspirated and added 150 μL DMSO to each well. The absorbance was determined at 540 nm, and relative cell viability rate was calculated.

### Clone formation assay

200 cells were plated in each well of a 6-well plate and cultured for two weeks in the cell culture incubator. Cells were incubated with 0.1% (w/v) crystal violet and preserved with 100% methanol. Representative fields were photographed, and the number of clones was counted.

### Plasmid DNA transfections

Cells at ~ 70% confluence were transfected with pLV-cDNA using Lipofectamine® 2000 regent, and incubated for 16 h. MTT and Western blot were conducted 24 h after plasmid transfection.

### Surface sensing of translation (SUnSET)

After treatment with puromycin (10 μg/mL) for 1 h, cells were lysed with lysis buffer and then centrifuged to extract the supernatant. Cellular protein was used for immunoprecipitation (IP), incubated with puromycin antibody. After incubation with protein A/G, the protein A/G-antigen–antibody complex was centrifuged and collected (George et al. 2021). After washing with PBST for 5 times, the antigen–antibody complex was eluted, followed by the determination of SREBP1 expression.

### UALCAN analysis

UALCAN database (http://ualcan.path.uab.edu/analysis.html) is a user-friendly and comprehensive web resource, which provides an easy way to obtain and analyze data from The Cancer Genome Atlas (TCGA) project (Chandrashekar et al. 2022). The mRNA expression of CTU2 in normal and tumor tissue, the mRNA expression of CTU2 at different stages of HCC progression (classified as normal and stage 1–4), the mRNA expression of CTU2 at different grades of HCC differentiation (classified as normal and grade 1–4), and the survival analysis of CTU2 expression and liver hepatocellular carcinoma (LIHC) patients were directly obtained from UALCAN database.

### Data analysis

The representative findings were shown, and each experiment was conducted at least three times. The error bar was displayed as mean ± SEM. Comparison of 2 groups used Student’s t-test. Comparison of > 2 groups used one-way or two-way ANOVA appropriately. If *P*-value < 0.05, the difference was deemed significant.

## Results

### CTU2 enhances proliferation of hepatoma carcinoma cells

**Fig. 1:**
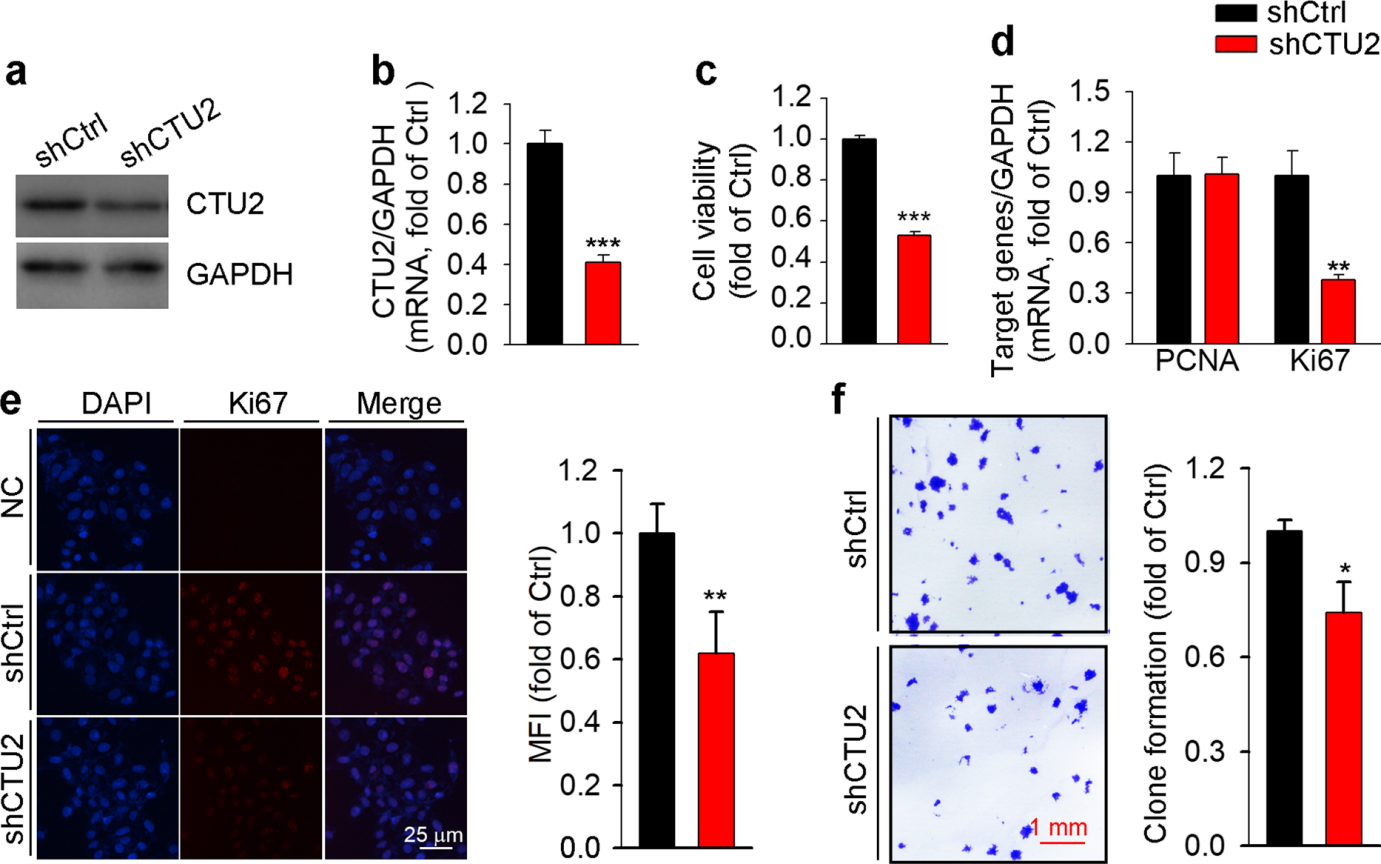
CTU2 expression regulates cell proliferation. HepG2 cells were transfected with shRNA-CTU2 (shCTU2) or shRNA-Control (shCtrl). The stable transfected cells were used to conduct the following assays: expression of CTU2 protein (**a**, *n* = 3), CTU2 mRNA (**b**, *n* = 3), and PCNA and Ki67 mRNA (**d**, *n* = 3) were determined by Western-blot and qRT-PCR, respectively. The effect of CTU2 knockdown on proliferation of HepG2 cells was determined by analysis of cell viability with MTT (**c**, *n* = 3) and clone formation (**f**, *n* = 3). Expression of Ki67 (**e**, *n* = 3) protein was determined by immunofluorescent staining with quantitative analysis of the mean fluorescent intensity (MFI). Two-tailed unpaired Student’s t-test was performed. *P < 0.05, **P < 0.01, ***P < 0.001 *vs*. shCtrl

A high level of CTU2 expression is observed in breast cancer and melanoma tissue and CTU2 promotes the development of subcutaneous breast tumor and melanoma tumor transplantation (Delaunay et al. 2016; Rapino et al. 2018). To determine the functions of CTU2, particularly on cell proliferation and tumorigenesis, we constructed CTU2 knockdown HepG2 cell line (shCTU2), and confirmed the manipulation of CTU2 expression in this cell line in protein (Fig. 1a) and mRNA (Fig. 1b) levels. To detect the effect of CTU2 expression on cell viability, both MTT and clone formation assays were conducted. We found cell viability was decreased by CTU2 knockdown in HepG2 cells (Fig. 1c). And inhibition of CTU2 expression reduced the clone-formation ability of HepG2 cells (Fig. 1f). At the molecular level, CTU2 knockdown reduced Ki67 mRNA with little effect on PCNA mRNA expression (Fig. 1d). In addition, the results of immunofluorescent staining showed that CTU2 knockdown repressed Ki67 protein expression (Fig. 1e). Therefore, Fig. 1 revealed that CTU2 knockdown can inhibit cell proliferation.

### CTU2 is an LXR target, and LXRβ plays a central role in modulating CTU2 expression

**Fig. 2:**
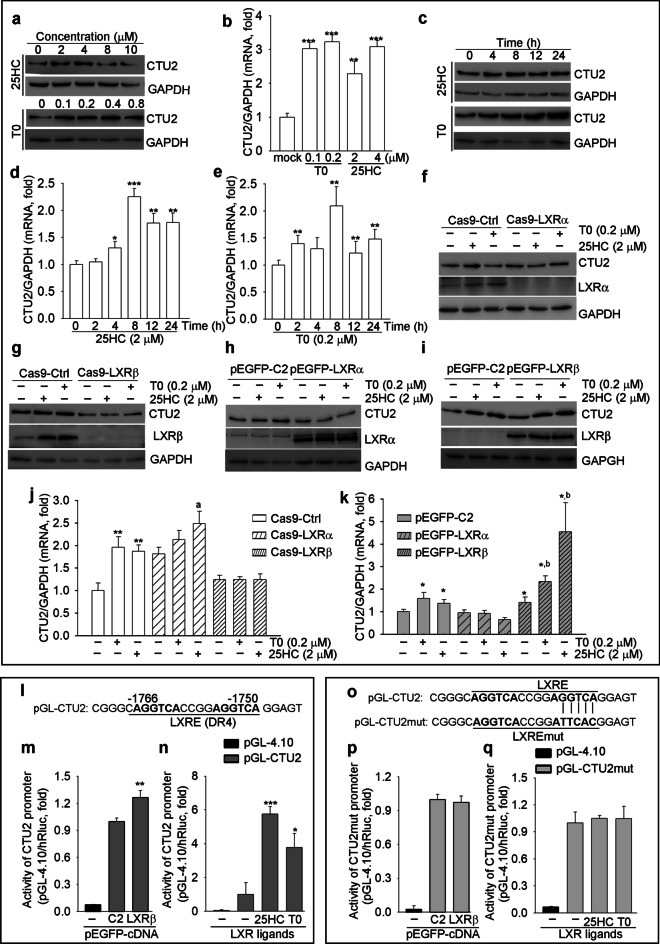
CTU2 is identified as an LXR target gene. HepG2 cells were treated with 25HC and T0901317 (T0) at the concentrations indicated for 16 h. Protein levels (**a**, *n* = 3) and mRNA levels (**b**, *n* = 3) of CTU2 were determined by Western blot and qRT-PCR; HepG2 cells were treated with 25HC (2 μM) and T0 (0.2 μM) for the time as indicated. Protein levels (**c**, *n* = 3) and mRNA levels (**d**, **e**, *n* = 3) were determined; Cas9-Ctrl, Cas9-LXRα and Cas9-LXRβ HepG2 cells were treated with 25HC (2 μM) and T0 (0.2 μM) for 16 h, and protein levels of CTU2, LXRα (**f**, *n* = 3) and CTU2, LXRβ (**g**, *n* = 3) and mRNA levels of CTU2 (**j**, *n* = 3) were determined; HepG2 cells were transfected with pEGFP-C2-LXRα or pEGFP-C2-LXRβ plasmid and treated with 25HC (2 μM) and T0 (0.2 μM) for 16 h. Protein levels of CTU2, LXRα (**h**, *n* = 3) and CTU2, LXRβ (**i**, *n* = 3), as well as mRNA levels of CTU2 (**k**, *n* = 3) were determined. One-way ANOVA (**b**, **d**, **e**) and two-way ANOVA (**j**, **k**) analysis was performed. *P < 0.05, **P < 0.01, ***P < 0.001 *vs.* HepG2, Cas9-Ctrl or pEGFP-C2 Ctrl group; ^a^*P* < 0.01 *vs.* pEGFP-LXRα Ctrl group; ^b^*P* < 0.01 *vs.* Cas9-LXRβ Ctrl group. **l**: the sequence of LXRE (letters in bold) in human CTU2 promoter (pGL-CTU2); **m, n**: HepG2 cells in 48-well plates were co-transfected with pEGFP-LXRβ (pEGFP-C2 for control) and pGL-CTU2 plasmid (10 ng/well) for 16 h (**m**, *n* = 3), or treated with LXR ligands (0.2 μM T0 or 4 μM 25HC) for 8 h after 16 h of pGL-CTU2 plasmid transfection (**n**, *n* = 3); **o**: the sequence for construction of human CTU2 promoter with mutated LXRE (LXREmut, pGL-CTU2mut); **p, q**: HepG2 cells in 48-well plates were co-transfected with pEGFP-LXRβ (pEGFP-C2 for control) and pGL-CTU2mut plasmid (10 ng/well) for 16 h (**p**, *n* = 3), or treated with LXR ligands (0.2 μM T0 or 4 μM 25HC) for 8 h after 16 h of pGL-CTU2 plasmid transfection (**q**, *n* = 3). CTU2 promoter activity (**m, n, p, q**) was determined by dual-luciferase reporter system. Two-way ANOVA analysis was performed. *P < 0.05, **P < 0.01, ***P < 0.001 *vs.* pGL-CTU2 mock group

The nuclear transcription regulatory element LXR, which is activated by ligands, is crucial for lipogenesis. It contains two isoforms, LXRα and LXRβ (Lee et al. 2014). Interestingly, we found LXR ligands, 25-hydroxycholesterol/25HC and T0901317/T0, induced CTU2 expression in a time and dose-dependent manner (Fig. 2a-e). Thus, we hypothesized that CTU2 expression might be regulated by LXR. We used CRISPR-Cas9 to construct LXRα (Cas9-LXRα) and LXRβ (Cas9-LXRβ) knockout HepG2 cells and corresponding control (Cas9-Ctrl) cells. Further, lack of LXRβ but not LXRα expression completely blocked LXR ligand-induced CTU2 mRNA and protein expression (Fig. 2f, g, j), indicating activation of CTU2 expression is in an LXRβ-dependent manner. Indeed, overexpression of LXRβ but not LXRα further enhanced LXR ligands-induced CTU2 protein expression (Fig. 2h, i, k).

By completing the sequence alignment assay, we found an LXR responsive element (LXRE) in CTU2 promoter with the sequence of AGGTCAN_4_AGGTCA which is 100% identical to the reserved LXRE (the 6 nucleotides are repeated and separated by any 4 nucleotides). Based on this sequence, we constructed a CTU2 promoter including LXRE (named as pGL-CTU2, Fig. 2l) and another CTU2 promoter with LXRE mutation (named as pGL-CTU2mut, Fig. 2o). As shown in Fig. 2m and 2n, CTU2 promoter activity was significantly induced by LXRβ overexpression or LXR ligand treatment. However, neither of LXRβ expression vector transfection nor LXR ligand treatment had effect of activity of the mutated CTU2 promoter (Fig. 2p, q). The results in Fig. 2 indicate that LXR activates CTU2 transcription, and LXRβ is the main isoform responsible for regulation of CTU2 expression.

### CTU2 activates lipogenesis to enhance cell proliferation

**Fig. 3:**
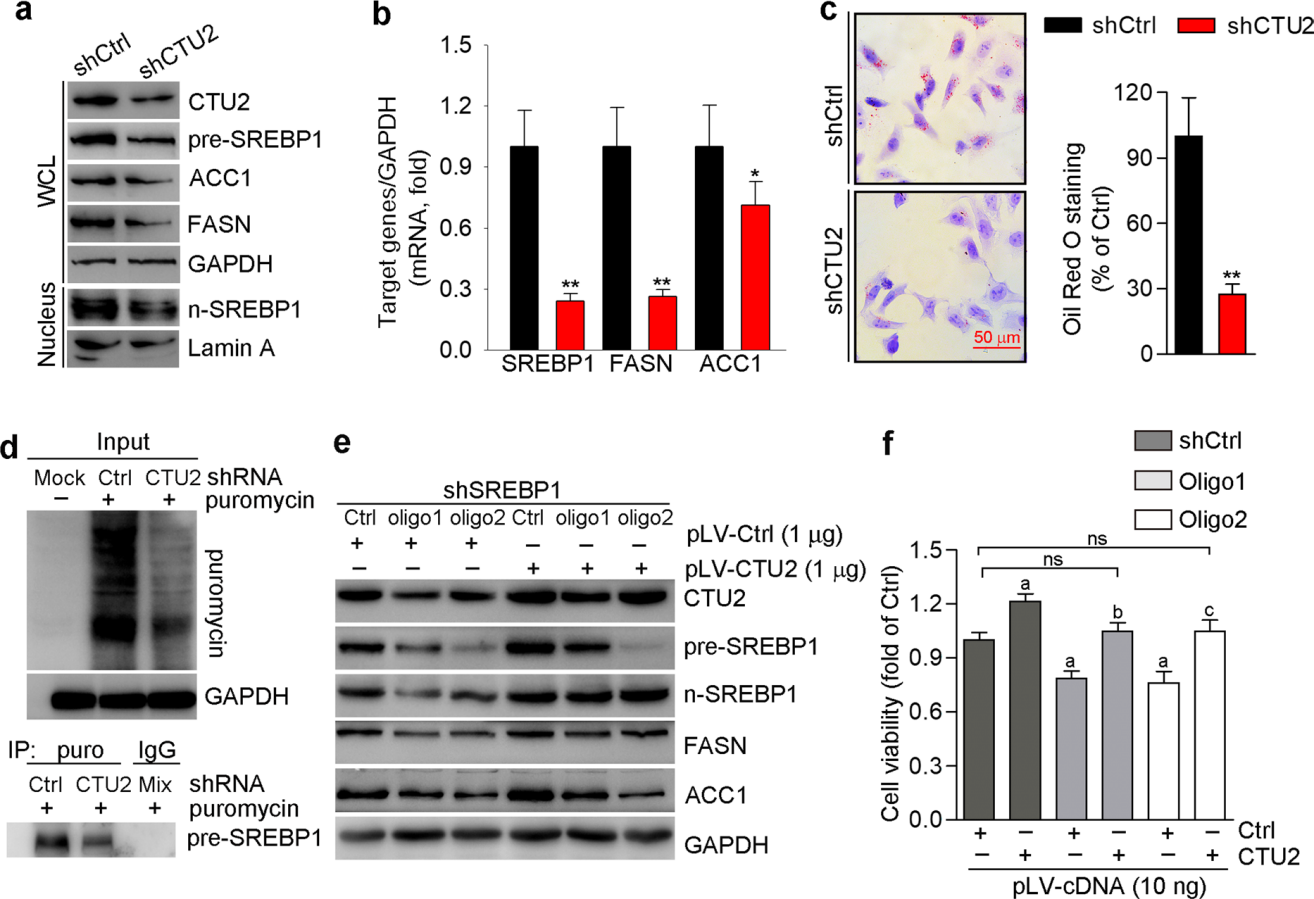
CTU2 activates cellular lipogenesis to enhance cell proliferation. shCtrl and shCTU2 HepG2 cells were used to conduct the following assays: Protein levels of CTU2, pre-SREBP1, ACC1, FASN, and n-SREBP1 in WCL (whole cell lysate) and nucleus were determined (**a**, *n* = 3), and mRNA levels of SREBP1, ACC1, and FASN were determined (**b**, *n* = 3). Lipid content was determined by Oil Red O staining (**c**, *n* = 3). Protein translational efficiency was determined by SUnSET assay (**d**, *n* = 3). *P < 0.05, **P < 0.01, ***P < 0.001 vs. shCtrl. shCtrl and shSREBP1 (targeted by oligo1 and oligo2)-transfected cells were transfected with pLV-Ctrl or pLV-CTU2 plasmid for 16 h. Cells were then used to determine CTU2, (pre/n)-SREBP1, FASN and ACC1 protein expression (**e**, *n* = 3) and cell viability (**f**, *n* = 3). ^a^*P* < 0.05 *vs*. shCtrl cells transfected with pLV-Ctrl, ^b/c^*P* < 0.05 *vs*. shSREBP1 (oligo1) or shSREBP1 (oligo2) cells transfected with pLV-Ctrl, ns: not significant. Two-tailed unpaired Student’s t-test (**b**, **c**) and two-way ANOVA (**f**) was performed

It is well-established that LXR is a key transcriptional element that regulates lipogenesis by inducing SREBP1, ACC1, and FASN expression. The de novo lipogenesis is important for rapidly proliferating cells. Lipids are also important signaling molecules for many biological processes including cell proliferation and survival (Rohrig and Schulze 2016). The activity of intracellular de novo lipogenesis is related to expression of lipogenic enzymes, such as ACC1 and FASN, which are regulated by the transcription factor SREBP1. To investigate the role of CTU2 in lipogenesis, we determined the effect of CTU2 knockdown on lipogenic gene expression. As shown in Fig. 3a, SREBP1 precursor form (pre-SREBP1), FASN, and ACC1 protein expression as well as SREBP1 maturation (SREBP1 nuclear form, n-SREBP1) were reduced by CTU2 knockdown. In addition, qPCR analysis showed that CTU2 knockdown decreased SREBP1, FASN, and ACC1 expression (Fig. 3b). The Oil Red O staining further confirmed the cellular lipid accumulation was reduced by CTU2 knockdown (Fig. 3c). Our results above indicated that CTU2 was deeply involved in the regulation of SREBP1, ACC1, and FASN expression, suggesting that CTU2 promoted expression of lipogenic genes to increase cellular lipid accumulation. To determine if CTU2 can affect translation efficiency of lipogenic genes, we conducted the SUnSET assay and found CTU2 knockdown repressed new synthesized SREBP1 protein (Fig. 3d).

To further determine the role of lipogenesis in CTU2-induced cell proliferation, we performed SREBP1 knockdown assay in HepG2 cells and then transfected the cells with pLV-CTU2 plasmids. Lipogenic genes (FASN and ACC1) expression was impaired by SREBP1 knockdown while reversed by high expressing CTU2 (Fig. 3e). Correspondingly, cell viability was repressed by shSREBP1 knockdown but reversed by CTU2 overexpression (Fig. 3f). These data further demonstrate that activation of cell proliferation by CTU2 is related to its lipogenic function.

### CTU2 knockdown enhances T0901317-inhibited tumor growth

**Fig. 4:**
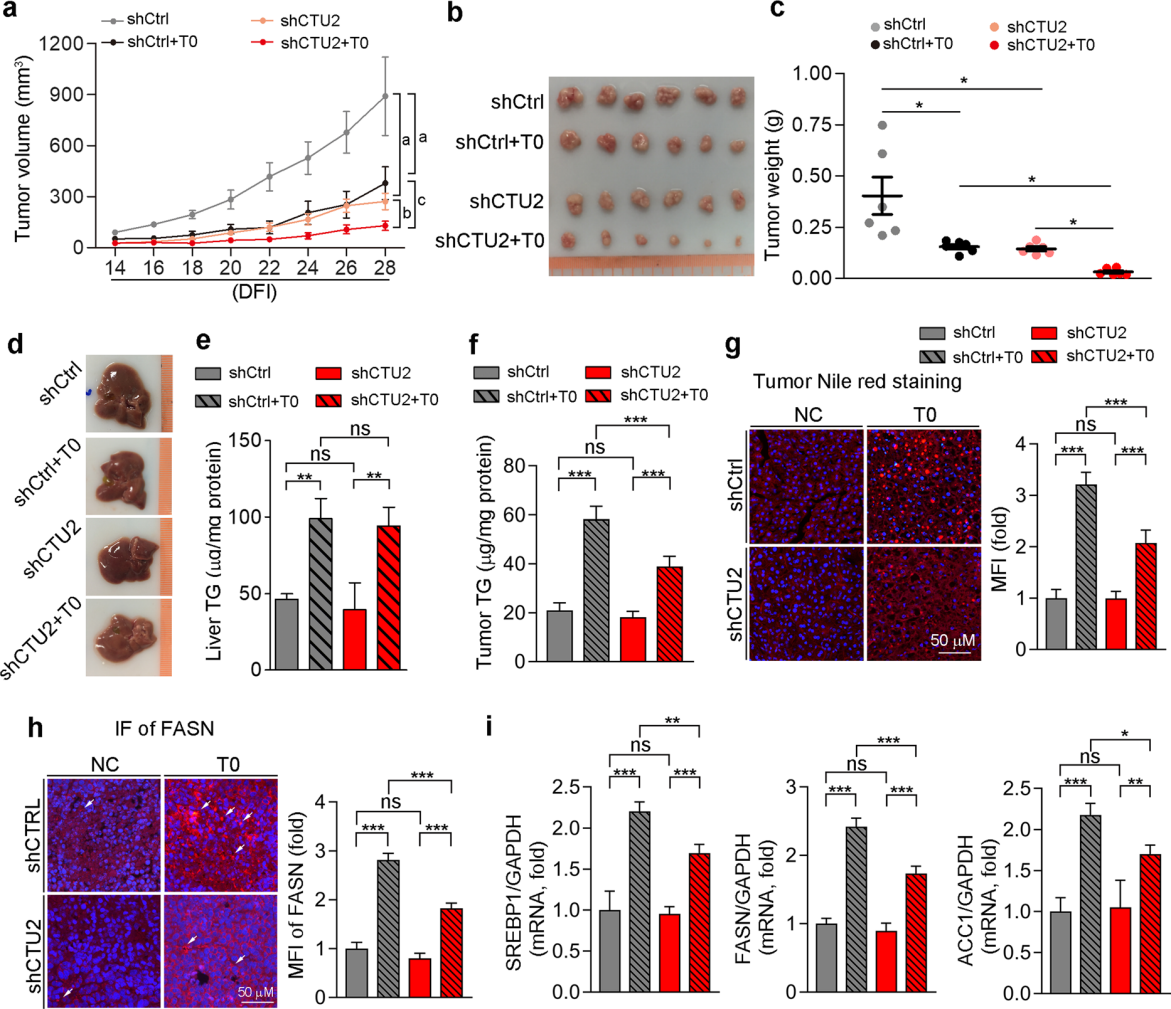
Inhibition of CTU2 expression synergizes the anti-tumor effect of T0901317. Nude mice were s.c. injected with shCtrl HepG2 or shCTU2 HepG2 cells (1 × 10^6^ cells/mouse). When tumors were visible, mice were divided into 2 groups (Ctrl/T0 group) respectively, which fed normal chow (NC) or normal chow containing T0 [5 mg/day/kg bodyweight (mpk)]. The tumor volumes were measured every other day (**a**). DFI: day following injection. At the end of experiment, mice were sacrificed followed by collecting livers and tumors. Tumors were photographed (**b**), weighed (**c**), and used for other assays. ^a^*P* < 0.05 *vs.* shCtrl, ^b^*P* < 0.05 *vs.* shCTU2, ^c^*P* < 0.05 *vs*. shCtrl + T0, *P < 0.05, (*n* = 6); livers were photographed (**d**) and used to determine triglyceride (TG) content (**e**). *P < 0.05, **P < 0.01, ns: not significant (*n* = 4); tumor tissue was used to determine TG levels (**f**), Nile red staining (**g**), FASN immunofluorescent (IF) staining (**h**), and SREBP1, FASN, and ACC1 mRNA levels (**i**). *P < 0.05, **P < 0.01, ***P < 0.001, ns: not significant, (*n* = 4). Two-way ANOVA was performed

LXR is a potential anti-tumor target, while its downstream CTU2 promotes cell proliferation in our in vitro study, indicating inhibition of CTU2 expression may enhance antitumorigenic effects of LXR. To determine it, we employed a human tumor xenograft mouse model by injecting HepG2 cells with CTU2 knockdown into nude mice subcutaneously. One week after cell injection, mice with visible tumors were started treatment of T0901317 (T0) contained in the normal chow. During the treatment, we routinely checked the tumor growth. As shown in Fig. 4a-c, the tumors formed by shCtrl HepG2 cells were three times bigger than the tumors generated from shCTU2 HepG2 cells. T0901317 was able to reduce tumor development, and CTU2 knockdown further reduced T0901317-inhibited tumor growth (Fig. 4a-c). Thus, inhibition of CTU2 expression can facilitate the inhibition of tumor growth by LXR ligand. Consistent with previous report, treatment of tumor-bearing mice with T0901317 induced lipid accumulation in the liver inevitably, while knocking down CTU2 in transplanted tumors had no effect on liver triglyceride levels (Fig. 4d-e) (Schultz et al. 2000). Meanwhile, knocking down CTU2 expression in HepG2 cells reduced triglyceride levels in tumor tissues under T0901317 treatment (Fig. 4f). The lipophilic dye Nile red staining showed that T0901317 increased lipid levels in tumor tissues, and CTU2 knockdown inhibited the increased lipid droplets induced by T0901317 (Fig. 4g). We also examined the expression of the lipogenic gene FASN in tumor tissues. T0901317 treatment elevated the protein level of FASN, and inhibiting CTU2 expression can antagonize the effect of T0901317 (Fig. 4h). And SREBP1, FASN, and ACC1 mRNA levels were decreased by CTU2 knockdown under T0901317 treatment (Fig. 4i). These results suggest that inhibiting CTU2 expression attenuates the lipogenic effects of LXR in tumor tissues and inhibiting CTU2 expression combined with T0901317 further represses tumor progression.

**Fig. 5:**
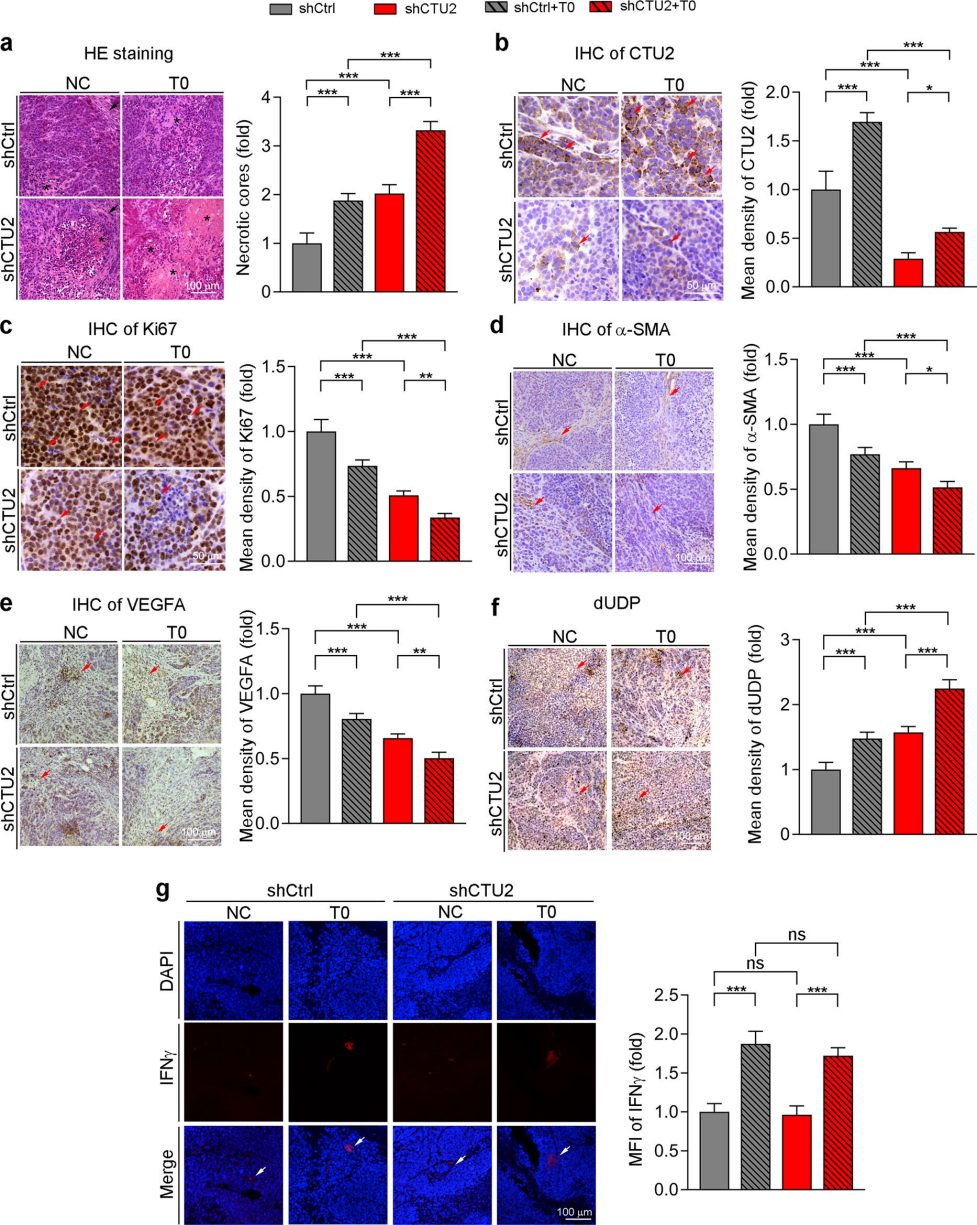
CTU2 promotes tumor cell proliferation, CAF formation and VEGFA expression, while T0901317 inhibits cell proliferation and induces cell apoptosis in vivo*.* At the end of experiment, tumors were collected from mice xenografted with shCtrl and shCTU2-transfected HepG2 cells. Tumor paraffin sections were prepared, followed by HE staining (**a**), IHC staining of CTU2 (**b**), Ki67 (**c**), αSMA (**d**), VEGFA (**e**), TUNEL staining (**f**), and IF staining of IFNγ (**g**). Black, red and white arrows indicate fibroblast-like cells, positive IHC and IF staining cells, respectively; * indicates necrotic cores. *P < 0.05, **P < 0.01, ***P < 0.001, ns: not significant, (*n* = 4). Two-way ANOVA was performed

To determine the related mechanism involved in CTU2-induced xenograft tumor growth and T0901317-mediated tumor inhibition, histopathological examination of tumor sections in each group was performed. As shown in Fig. 5a, the tumor sections in different groups presented different kinds of histological characteristics. CTU2 IHC staining results showed that tumor sections in shCtrl HepG2 cell-injected groups presented more CTU2 positive staining cells, implying that tumor-forming cells retained the genotype at the time of inoculation (Fig. 5b). Ki67 IHC staining showed that higher CTU2 expression was associated with more Ki67 positive staining cells, while T0901317 treatment decreased Ki67 positive cells, and inhibiting CTU2 expression can synergize the effects of T0901317 consistent with qRT-PCR results (Fig. 5c; Fig. S1).

We found there were more fibroblast-like cells in tumor sections of shCtrl cell injection group by HE staining (Fig. 5a, black arrows), and identified these cells as fibroblasts by determination of αSMA protein and mRNA levels (Fig. 5d; Fig. S1). Since we observed more fibroblasts in the sections of faster-growing tumor, we hypothesized that these cells might be cancer-associated fibroblasts (CAFs). CAFs are important components of tumor microenvironment, which result in the stroma support growth and invasion of epithelial cells (Calon et al. 2012). CAFs can secrete more cytokines than normal fibroblasts, such as vascular endothelial growth factor A (VEGFA) (Inoue et al. 2019). VEGFA is highly expressed during tumor angiogenesis and closely related to tumor angiogenesis (Fodor et al. 2019). Herein we found inhibition of CTU2 expression decreased VEGFA protein and mRNA expression (Fig. 5e; Fig. S1), which indicated that CTU2 may promote CAF presentation and take part in tumor vascular development.

LXR agonist is believed to kill tumor cells by activating tumor cells apoptosis. Indeed, T0901317 treatment caused more tumor necrosis as shown by HE staining (Fig. 5a, asterisk), and TUNEL staining further confirmed that T0901317 treatment induced cell apoptosis in transplanted tumors (Fig. 5f). We previously found LXR agonist was able to promote the expression and secretion of IFNγ, and exert the anti-tumor efficiency (Wang et al. 2016). IFNγ is also believed to induce hepatoma carcinoma cell apoptosis (McCullough et al. 2006). Therefore, we hypothesized that LXR agonist-induced apoptosis of tumor cells might be related to IFNγ expression. Indeed, we found IFNγ protein and mRNA levels was obviously induced by T0901317 treatment while CTU2 has no effect on IFNγ expression (Fig. 5g; Fig. S1). Taken together, the results in Fig. 5 demonstrated that CTU2 knockdown inhibited cell proliferation, tumor vascular generation and CAF formation to hinder tumor progress, while T0901317 inhibited cell proliferation and induced cell apoptosis to inhibit tumor development. Inhibiting CTU2 expression could synergize with the anti-tumor effects of T0901317.

### CTU2 is up-regulated in HCC samples

**Fig. 6:**
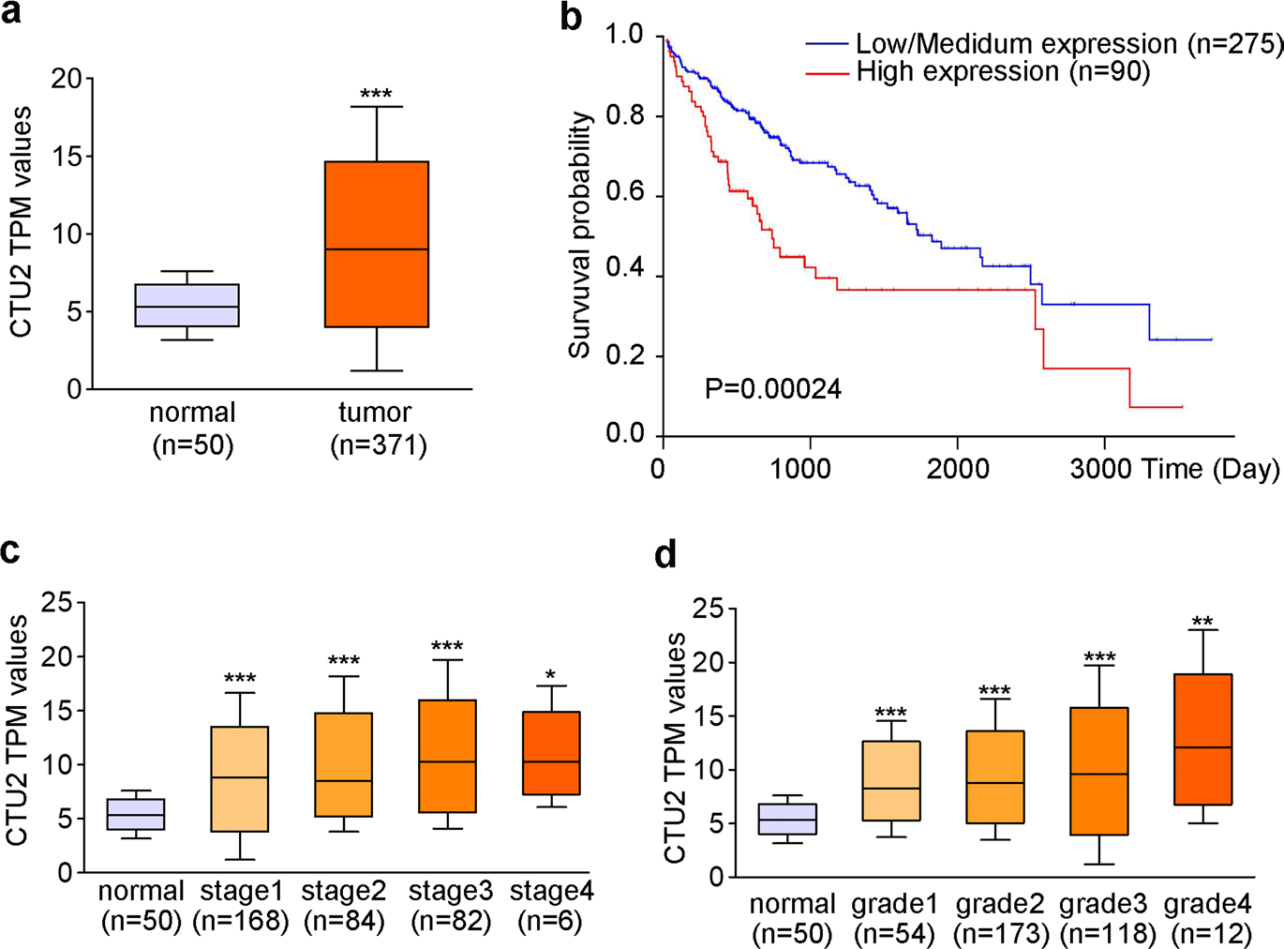
CTU2 expression is elevated in human HCC development. **a**: CTU2 mRNA expression in normal (*n* = 50) and HCC (*n* = 371) samples from the UALCAN database; **b**: Km-plot analysis of CTU2 expression and survival of HCC patients consist of 275 patients in CTU2 low/medium expression group and 90 patients in CTU2 high expression group from the UALCAN database; **c**: the relative expression of CTU2 mRNA in normal (*n* = 50), stage 1 (*n* = 168), stage 2 (*n* = 84), stage 3 (*n* = 82), and stage 4 HCC (*n* = 6) samples from the UALCAN database; **d**: the relative expression of CTU2 mRNA in normal (*n* = 50), grade 1 (*n* = 54), grade 2 (*n* = 173), grade 3 (*n* = 118), and grade 4 HCC (*n* = 12) samples from the UALCAN database; TPM: Transcript per million. Two-tailed unpaired Student’s t-test **a** paired Log Rank Test **b** and one-way ANOVA **c, d** were performed. *P < 0.05, **P < 0.01, ***P < 0.001 *vs.* normal

To determine whether CTU2 plays a role in clinical HCC development, we retrieved the gene expression data of CTU2 from the UALCAN database (https://ualcan.path.uab.edu/index.html). CTU2 was up-regulated in HCC tumor compared to normal tissue (Fig. 6a). There was a negative correlation between CTU2 expression and the survival rate of HCC patients (Fig. 6b). CTU2 levels were positively correlated to tumor progression (Fig. 6c, classified as normal and stage 1–4) and poor differentiation (Fig. 6d, classified as normal and grade 1–4). These results suggested that up-regulated CTU2 predicted aggressive clinical features and poor survival probability of HCC patients.

## Discussion

CTU2 along with the conserved CTU1 protein takes part in the 2-thiolation of cytosolic tRNAs. The tRNA thiolation together with mcm5S2U is proposed to facilitate mRNA and tRNA codon reading accuracy during mRNA translation (Ikeuchi et al. 2006). mRNA translation is a major step in the gene expression. Dysregulation of mRNA translation have been observed in a variety of human malignancies, including HCC (Silvera et al. 2010). We revealed the role of CTU2 in HCC growth. We observed CTU2, which is an LXR target, took part in fatty acid synthesis and promoted cell proliferation.

A tight control of cell growth is essential for the maintenance of homeostasis in healthy organs and normal tissues. Failures in the control of cell growth result in unchecked cell divisions and cancer development. CTU1 is involved in cell growth control in certain types of human cancer (Yousef et al. 2004). Herein we determined the role of CTU2 in cell growth. We found CTU2 knockdown inhibited HepG2 cells proliferation (Fig. 1c-f), indicating that CTU2 may participate in HCC development. Correspondingly, CTU2 knockdown impaired tumor formation in vivo (Fig. 4a-c). Mechanistically, CTU2 knockdown inhibited tumor cell proliferation, CAF presentation, and tumor vascular generation (Fig. 5c-e) in vivo.

LXR and its ligands are considered as potential therapeutic targets against cancer. LXR ligands are able to regulate cell cycle proliferation and induce cancer cells apoptosis. We have reported that T0901317 blocked transplanted lung carcinoma growth by stimulating IFN-γ expression (Wang et al. 2014). T0901317 can also block TGFβ signaling thereby reducing CAFs differentiation and HCC development (Morén et al. 2019). There is also evidence that the LXR agonist T0901317 can inhibit melanoma growth by promoting melanoma cell apoptosis through the caspase3-pathway (Zhang et al. 2014). T0901317 can also inhibit tumor cell proliferation through the PPARγ-LXRα-ABCA1 pathway (Yang et al. 2012). Withaferin A was found to be dual activator of LXR and FXR, and Withaferin A exerted anti-tumor effects without inducing hepatic lipid accumulation, suggesting the important role of LXR agonists in anti-tumor (Shiragannavar et al. 2020, 2023). In this study, LXR activation also resulted in inhibition of transplanted tumor growth (Fig. 4a-c). Mechanistically, T0901317 inhibited cell proliferation and promoted cell apoptosis by inducing IFNγ expression (Fig. 5c, f, and g). In addition, our study also showed that inhibiting CTU2 expression can suppress tumor cell proliferation (Fig. 5c), CAF presentation (Fig. 5d, e), and promote HCC tumor apoptosis (Fig. 5f) in tumor-bearing mice model. Therefore, inhibition of CTU2 expression synergizes with T0901317 to suppress HCC growth in tumor-bearing mice (Fig. 4a-c).

Increased de novo lipid synthesis can provide important components of membrane structure, store energy, and play an important role in AKT signaling to control cell proliferation through synthesizing second messengers for rapidly proliferating cancer cells (Rohrig and Schulze 2016). In this study, CTU2 knockdown decreased SREBP1, FASN, and ACC1 expression and lipid accumulation in cells (Fig. 3a-c). As one of the important tRNA thiolation enzymes guaranteeing efficient translation, CTU2 directly promoted the translation of SREBP1 (Fig. 3d). Besides, CTU2 overexpression could reverse the cell growth inhibition induced by SREBP1 knockdown (Fig. 3f), suggesting the importance of CTU2-induced lipid synthesis in cell proliferation. However, CTU2 overexpression still promoted cell proliferation in SREBP1 knockdown cells. Interestingly, SREBP1 shRNA decreased pre-SREBP1 protein levels without affecting nuclear SREBP1 protein levels (n-SREBP1, the active form of SREBP1) in pLV-CTU2 transfected HepG2 cells. In this way, CTU2 may promote cell proliferation by promoting the nuclear transfer of SREBP1 in shSREBP1 cells. In addition, we found that CTU2 knockdown decreased SREBP1, ACC1, and FASN expression at the mRNA levels. The process of lipid metabolism is multivariate and feedback-driven. The LXR pathway can also be regulated by other lipid metabolism signaling, such as the SREBP2 and FXR pathways. For instance, FXR was found to inhibit the LXRα-SREBP1 pathway with decreasing SREBP1 mRNA levels (Watanabe et al. 2004). There are currently few studies on CTU2, and the role of CTU2 in lipid metabolism remains unknown. Our study found that CTU2 may be a potential target in the lipid metabolism process. Other roles of CTU2 in lipid metabolism are still unknown. CTU2 may affect the function of LXR through other regulators, thereby affecting the target genes of LXR at the mRNA level.

LXRβ, but not LXRα, and synthetic ligands could activate CTU2 expression (Fig. 2a-k). We found an LXR-responsive element in the CTU2 promoter (Fig. 2l-q), indicating LXR is involved in development of HCC by regulating the expression of CTU2. Even though T0901317 suppresses tumor growth by promoting apoptosis and slowing down proliferation, its side effects of promoting lipogenesis hinder the application of T0901317 (Chisholm et al. 2003). Our study found that LXR can transcriptionally activate CTU2, thereby promoting protein synthesis of lipogenic genes. And inhibiting the expression of CTU2 can inhibit the protein synthesis of lipogenic genes. Therefore, inhibiting CTU2 and T0901317 in combination can suppress the side effects of T0901317 promoting lipogenesis in tumor tissues. Interestingly, reduction of CTU2 expression in HepG2 cells had no effect on liver TG levels in xenograft nude mice model (Fig. 4e). However, inhibiting CTU2 expression reduced TG levels in tumor tissues (Fig. 4f, g). It is necessary to use CTU2 knockout mice to explore the role of CTU2 in the LXR-mediated lipid metabolism and anti-tumor pathway.

By comparing tumor burden in each group, we found tumors generated from CTU2 knockdown cells with T0901317 treatment were the smallest (Fig. 4a), indicating that blocking CTU2 may improve the anti-tumor effect of LXR ligands. CTU2 was significantly up-regulated in human HCC tumor compared to normal tissue and tightly associated with tumor development (Fig. 6a-d).

In summary, we demonstrate that CTU2, which is an LXR target, enhances lipid synthesis. The elevated lipid synthesis is of great importance in CTU2-induced cell proliferation. CTU2 is also able to facilitate CAF formation and tumor vascular generation in vivo. CTU2 inhibition may improve the anti-tumor effect while relief the side effect of LXR ligands. However, we should use CTU2 liver-specific knockout mice for verification in future work, further exploring the mechanism of CTU2 regulating HCC and determining that CTU2 is an effective target for the treatment of HCC.

## Conclusions

CTU2 enhances proliferation of hepatoma carcinoma cells. Mechanically, CTU2 is a target gene of LXR, and inhibition of CTU2 expression can synergize the pro-apoptosis and anti-proliferation effects of LXR agonist in tumors. Moreover, inhibition of CTU2 expression can inhibit cell proliferation by decreasing lipogenesis, directly reducing the synthesis of proteins involved in lipogenesis. Meanwhile, inhibition of CTU2 also reduced angiogenesis by reducing CAF. Hence, inhibition of CTU2 expression in HepG2 cells represses tumor growth in vivo*.* Our findings define CTU2 as a promising target for HCC treatment and provide a novel strategy for the clinical use of LXR agonists in HCC treatment.

## Supplementary Information

Below is the link to the electronic supplementary material.

Supplementary file1 (DOCX 176 KB)

## Data Availability

The data in this article will be shared on reasonable request to the corresponding author.
