# Supplementary material for: Activation of CTU2 expression by LXR promotes the development of hepatocellular carcinoma
Source: Cell Biol Toxicol. 2024 Apr 17;40(1):23. doi: 10.1007/s10565-024-09862-9 (PMC11024035; doi:10.1007/s10565-024-09862-9)
Supplement: Supplementary file 1 — Supplementary file1 (DOCX 176 KB) [file 10565_2024_9862_MOESM1_ESM.docx]

Supplementary Table S1. Sequence of the oligos for vector construction (5’→3’)

| Oligo | Sequence (5’→3’) |
| --- | --- |
| pLV-CTU2 forward | GCTCTAGAGCCACCATGTGTCAGGTGGGCGAGG |
| pLV-CTU2 reverse | CGGCTAGCTCAGCTCTGGCCCGCCTCGTCGTCA |
| shCTU2 sense | CCGGGCAGAAGTGTGTGAAGTGCAACTCG  AGTTGCACTTCACACACTTCTGCTTTTTG |
| shCTU2 anti-sense | AATTCAAAAAGCAGAAGTGTGTGAAGTGC  AACTCGAGTTGCACTTCACACACTTCTGC |
| shSREBP1 Oligo1 sense | CCGGAAGCTGAATAAATCTGCTGTCCTCG  AGGACAGCAGATTTATTCAGCTTTTTTTG |
| shSREBP1 Oligo1 anti-sense | AATTCAAAAAAAGCTGAATAAATCTGCTGTC CTCGAGGACAGCAGATTTATTCAGCTT |
| shSREBP1 Oligo2 sense | CCGGAACCAGAAACTCAAGCAGGAGTCG  AGCTCCTGCTTGAGTTTCTGGTTTTTTTG |
| shSREBP1 Oligo2 anti-sense | AATTCAAAAAAACCAGAAACTCAAGCAGGA  GCTCGAGCTCCTGCTTGAGTTTCTGGTT |
| LXRα sgRNA | TCGGCTTCGCAAATGCCGTC |
| LXRβ sgRNA | ACCCCGGCAGGCATAGCGCC |
| pGL-CTU2 sense | AGGCTAGCAAGATGCTCCACAATTCCATG |
| pGL-CTU2 anti-sense | ATAGATCTTAGT CCTCGCCCACCTGAC |
| pGL-CTU2mut sense | GCAGGTCACCGGATTCACGGAGTTCGAGAC |
| pGL-CTU2mut anti-sense | GTGAATCCGGTGACCTGCCCGTCTCAGCCT |

Supplementary Table S2. Sequence of the primers for qRT-PCR analysis (5’→3’)

| Gene (human) | Forward | reverse |
| --- | --- | --- |
| CTU2 | TCATCTTTGTTGACGAGGGA | GCTTGCAGAATGGGCTTC |
| GAPDH | TGATGACATCAAGAAGGTGGTGAAG | TCCTTGGAGGCCATGTGGGCCAT |
| Ki67 | TGACTTCCTTCCATTCTGAAGAC | TGGGTCTGTTATTGATGAGCC |
| PCNA | AAGAGAGTGGAGTGGCTTTTG | TGTCGATAAAGAGGAGGAAGC |
| SREBP1 | GGATTGCACTTTCGAAGACATG | AGCATAGGGTGGGTCAATAGG |
| FASN  ACC1 | AACTCCAAGGACACAGTCACCAT  GCCATTGGGATTGGGGCTTAC | CAGCTGCTCCACGAACTCAAA  CCCGCCCGAGGACTTTGTTG |
| α-SMA | GTGCGATGACGTGATCTGTGA | CGGTGGTTTCTTGGTCGGT |
| VEGFA | AGGGCAGAATCATCACGAAGT | AGGGTCTCGATTGGATGGCA |
| IFNγ | TCGGTAACTGACTTGAATGTCCA | TCGCTTCCCTGTTTTAGCTGC |

Supplementary Table S3. Antibodies information

| Target antigen | Source | Catalog # |
| --- | --- | --- |
| CTU2 | Abcam, USA | ab177160 |
| Pre-SREBP1 | Proteintech, USA | 14088-1-AP |
| Lamin A | Proteintech, USA | 10298-1-AP |
| n-SREBP1 | Nuvos, USA | NB100-2215 |
| FASN | Santa Cruz, USA | sc-48357 |
| Ki67 | Santa Cruz, USA | sc-23900 |
| PCNA | Santa Cruz, USA | sc-56 |
| GAPDH | Santa Cruz, USA | sc-365062 |
| ACC1 | Cell Signaling Technology  USA | 3676 |
| puromycin | Merck Millipore, UK | MABE343-AF488 |
| FASN | Abclonal, China | A0461 |

**Supplementary figure legends**

**
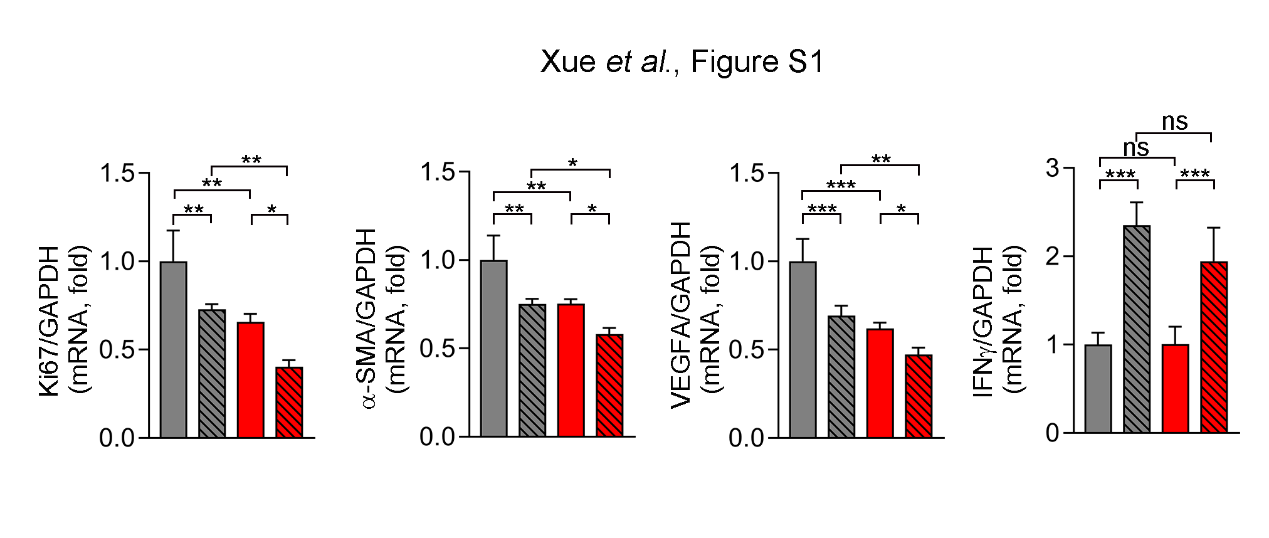
**

**Fig S1.** CTU2 decreased Ki67, α-SMA, and VEGFA mRNA levels without affecting IFNγ expression

mRNA levels of Ki67, α-SMA, VEGFA, and IFNγ in tumor tissues were determined by qRT-PCR. **P*<0.05, ***P*<0.01, ****P*<0.001, ns: not significant, (*n*=4). Two-way ANOVA was performed.
